# Matching Faces Against the Clock

**DOI:** 10.1177/2041669516672219

**Published:** 2016-10-03

**Authors:** Markus Bindemann, Matthew Fysh, Katie Cross, Rebecca Watts

**Affiliations:** School of Psychology, University of Kent, Canterbury, UK

**Keywords:** face matching, time pressure, response bias

## Abstract

This study examined the effect of time pressure on face-matching accuracy. Across two experiments, observers decided whether pairs of faces depict one person or different people. Time pressure was exerted via two additional displays, which were constantly updated to inform observers on whether they were on track to meet or miss a time target. In this paradigm, faces were matched under increasing or decreasing (Experiment 1) and constant time pressure (Experiment 2), which varied from 10 to 2 seconds. In both experiments, time pressure reduced accuracy, but the point at which this declined varied from 8 to 2 seconds. A separate match response bias was found, which developed over the course of the experiments. These results indicate that both time pressure and the repetitive nature of face matching are detrimental to performance.

## Introduction

In forensic face matching, observers must decide whether pairs of unfamiliar faces depict one person (i.e., an identity match) or two different people (an identity mismatch). This task is of considerable applied importance. Person identification at borders and national airports, for example, relies on the routine matching of face photographs in passports to their bearers. A key purpose of this task is to detect impostors, who attempt to evade detection by travelling under the valid identity documents of another person of similar appearance. Such real-life identity mismatches are a documented security concern (see, e.g., CPNI, 2007; FRONTEX, 2012; Stevens, 2011), but the scale of this problem remains unknown. Laboratory studies of face matching have therefore been instrumental in estimating the accuracy of person identification in these settings, and in understanding why such errors might arise.

This research has demonstrated consistently that unfamiliar face matching is error prone. Under highly optimized conditions, in which observers have to match same-day, high-quality photographs of frontal faces with a neutral expression, 10% to 20% errors are routinely made. This level of performance is already considered problematic for applied settings (see Jenkins & Burton, 2008; Robertson, Middleton, & Burton, 2015), but deteriorates further under more realistic conditions, such as when photo-identity documents are used (Bindemann & Sandford, 2011; Kemp, Towell, & Pike, 2013), photo quality is degraded (Bindemann, Leach, Attard, & Johnston, 2013), and to-be-matched photographs are taken many months apart (Megreya, Sandford, & Burton, 2013). This indicates that person identification from passports, which are typically valid through a 10-year period, is particularly challenging. Such problems are likely to be compounded in operational settings by the repetitive nature of identification tasks, which also reduces accuracy dramatically (Alenezi & Bindemann, 2013; Alenezi, Bindemann, Fysh, & Johnston, 2015). The accuracy of passport officers also appears to be as error prone as that of untrained student participants, to the point that some of these professionals perform at near-chance levels despite extensive work experience (White et al., 2014; but see White, Dunn, Schmid, & Kemp, 2015; White, Phillips, Hahn, Hill, & O’Toole, 2015). Overall, these findings therefore raise substantial concern about face matching as a reliable means of person identification in applied settings.

In this study, we investigate a factor that has received limited attention so far, but is also important practically. In laboratory studies on face matching, observers are typically given unlimited time to complete the task to measure best-possible accuracy (see, e.g., Bindemann, Avetisyan, & Rakow, 2012; Burton, White, & McNeill, 2010; Henderson, Bruce, & Burton, 2001; Megreya & Bindemann, 2015; Megreya & Burton, 2006). This differs from applied settings, in which observers are under time pressure to complete this task. In the United Kingdom, for example, 95% of passengers from the European Economic Area (EEA) must be processed through immigration within 25 minutes of arrival. While it is difficult to determine how much time this leaves available to process individual travellers, reports indicate that passenger numbers often exceed processing capacity (see, e.g., Home Affairs Committee, 2012; Independent Chief Inspector of Borders and Immigration [ICI], 2014, 2015). This suggests that passport officers frequently experience time pressure to perform this task.

So far, only limited research has systematically explored the effect of time pressure on face-matching accuracy. In one study, observers matched pairs of faces within a 6- or 15-second time window, the remaining time of which was indicated by a moving time bar above the face stimuli (Lee, Vast, & Butavicius, 2006). In this setup, an effect of time pressure was obtained only when observers were asked to check additional semantic information, such as a person’s age, height, or postal address, alongside the face. This indicates that unfamiliar faces can be matched within a 6-second limit and is consistent with self-paced laboratory studies, which consistently reveal average response times of less than 6 seconds in comparable tasks (see, e.g., Estudillo & Bindemann, 2014; Moore & Johnston, 2013; Papesh & Goldinger, 2014).

In a more recent study, observers were asked to match faces while display time was limited to several much shorter durations, ranging from 200 milliseconds to 2 seconds (Özbek & Bindemann, 2011). In this experiment, accuracy was highest in the 2-second condition and comparable to when no viewing time limits were imposed. However, although this indicates that 2 seconds might be sufficient for face matching, participants’ responses were recorded after the faces were removed from view, and response speed was not emphasized as a task requirement. Consequently, the data from this study cannot adequately capture the full duration of the face-matching process.

In addition, a very recent study also suggests that accuracy is affected by time pressure (White, Phillips, et al., 2015). In this study, accuracy declined when observers viewed face pairs for 2 seconds compared to 30 seconds before recording their matching decisions. However, the large range between viewing time limits (2 seconds vs. 30 seconds) makes it impossible to determine a more precise cut-off point at which accuracy begins to decline. Moreover, the 2-second displays were always presented first and the stimuli were repeated across conditions, leaving open the possibility that practice effects can account for these results.

All of these studies also fail to capture another aspect that is relevant to understanding time pressure in applied settings. In these studies, viewing time limits were decided a priori and administered on a trial-by-trial basis (e.g., this could be 200, 500, 1,000, or 2,000 milliseconds in Özbek & Bindemann, 2011). In operational settings, time pressure applies to more extended periods that encompass many trials. As a consequence, some faces can be given proportionately more attention if this is required by an observer to make a decision. If overall time limits allow, any lost time can then be recouped during subsequent identifications.

In this study, we report two experiments that manipulated time pressure in this way, to assess its effect on face-matching accuracy. For this purpose, two onscreen displays were presented that constantly updated to inform observers about (a) the numbers of faces that still needed to be processed within a given time frame and (b) whether they were on track to meet or miss a time target for processing these faces. In Experiment 1, we employed this novel paradigm under conditions in which the average time limit for matching faces decreases or increases systematically across blocks from 10 to 2 seconds. Experiment 2 then explored performance when these time limits remain constant across blocks.

## Experiment 1

In this experiment, observers matched pairs of faces across five blocks. Within each block, time pressure was implemented with a queue index to inform participants of the number of stimuli that remained to be processed, while a second display provided feedback on whether they were on track to meet time targets for processing these faces. This setup administers time pressure flexibly, so that slow responses during some identification decisions can be compensated for by faster responses on other trials, provided that sufficient time is left available to complete the task overall. In a between-subjects design, time pressure gradually increased across blocks, by reducing the average time available to match faces from 10 to 2 seconds, or decreased in reverse order. This design should reveal at which time limit face-matching accuracy begins to decline. In turn, this should inform how much time is required on average to achieve best possible accuracy in face matching.

## Method

### Participants

A total of 40 undergraduate students from the University of Kent participated in this experiment in exchange for course credit or a small fee. Of these, 20 observers participated under increasing time pressure (19 women, 1 man; with a mean age of 21.1 years) and 20 under decreasing time pressure (19 women, 1 man; with a mean age of 20.4 years). All reported normal or corrected-to-normal vision. Sample size was comparable to previous studies in this field (see, e.g., Lee et al., 2006; Özbek & Bindemann, 2011; White, Phillips, et al., 2015).

### Stimuli

The stimuli consisted of 200 pairs of White faces (100 women), which were drawn from the Glasgow University Face Database (see Burton et al., 2010). One face in each pair was photographed using a digital camera, whereas the other was a still image extracted from high-quality video. Each face was depicted from the front, while bearing a neutral expression, and was presented in grayscale. Additionally, faces were presented side by side, at a width of 350 pixels, and a screen resolution of 72 ppi. Half of these pairs depicted the same person (an identity match), while the remainder portrayed two different individuals (a mismatch).

**Figure 1.**
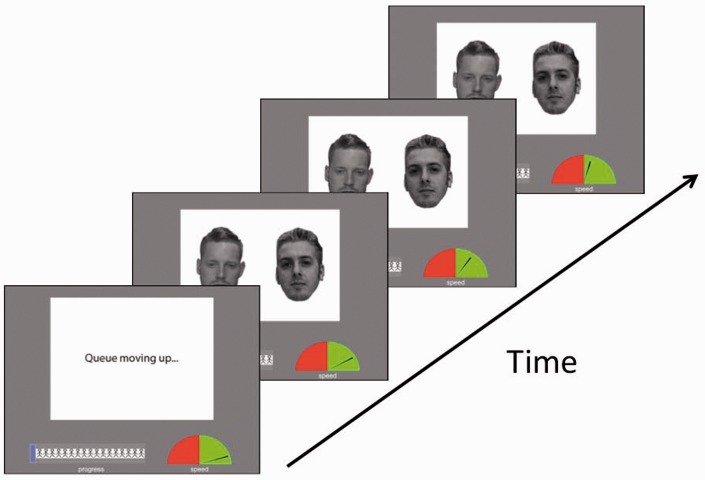
An illustration of the stimulus displays and trial procedure. A progress bar informed observers about the number of faces that still needed to be processed, while a speed gauge indicated whether they were on track to meet the time targets.

Time pressure was implemented via two additional onscreen displays, which were presented below the face stimuli (for an illustration, see Figure 1). One of these displays was a queue index that informed participants of the number of stimuli that remained to be processed in a block of trials. This display consisted of a row of person icons, to represent a queue of people, and a superimposed progress bar, which gradually filled in with each completed trial. The second display provided feedback on whether participants were on track to meet a time target for processing these faces. This display was composed of equally sized green and red zones, which acted as a time index. A needle was presented in these zones, depending on whether participants were within a given time target (green zone) or failing to meet the target time (red zone). The location of the needle within the speed display was updated every 100 milliseconds, while the extent to which the needle penetrated into each of the zones, was proportional to how far participants were ahead or behind the target time. This was based on the average speed of their responses, calculated across the completed trials in a block, in comparison to the same number of trials multiplied by the set mean time target (i.e., 2, 4, 6, 8, or 10 seconds). The displays were reset at the beginning of each block.

### Procedure

The experiment was run using *PsychoPy* software (Peirce, 2007). On each trial, participants were presented first with a one-second display with the message “Queue moving up …” to indicate that a face pair was about to appear. This was followed by a stimulus display, which remained onscreen until a response was registered. Participants were asked to decide as accurately as possible whether the face pair presented on each trial consisted of an identity match or mismatch by pressing one of two possible response keys on a standard computer keyboard.

Participants completed 200 trials, presented across five blocks of 40 face pairs (20 identity matches and 20 mismatches). The stimuli that appeared in each of these blocks were counterbalanced across participants over the course of the experiment. The average time to complete each trial was adjusted across blocks. In the increasing time-pressure condition, 10 seconds were allowed on average per face pair in Block 1, 8 seconds in Block 2, 6 seconds in Block 3, 4 seconds in Block 4, and 2 seconds in Block 5. In the decreasing time-pressure condition, the same time limits were applied, but pressure decreased across blocks, ranging from 2 seconds in Block 1 to 10 seconds in Block 5.

The speed display below the face stimuli was programmed to reflect these time limits for each block, while the queue display was updated on completion of each trial. Participants were briefed about these displays at the beginning of the experiment and were instructed to utilize these to adjust their response speed accordingly. Specifically, participants were informed that their speed could drop into the red zone on the speed dial if more time was required for some identifications. However, they were also required to monitor the queue display and adjust their response times so that the needle on the speed dial was (back) in the green zone by the end of each block. Participants were not told in advance that time pressure would increase or decrease across blocks.

## Results

### Response Times

**Figure 2.**
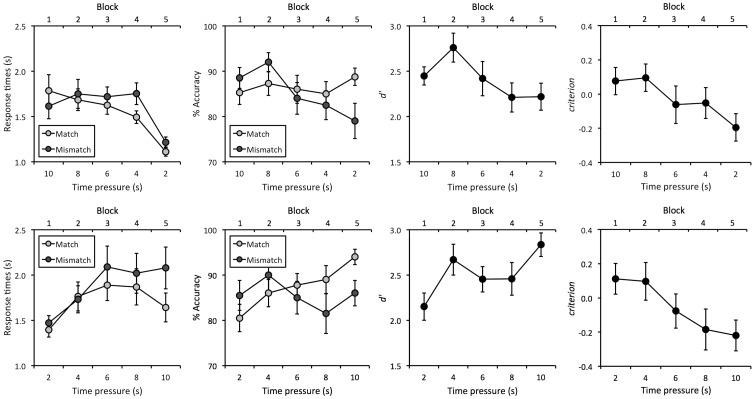
Response times (in seconds), face-matching accuracy (in percentage), *d′* and *criterion* for the increasing (top row) and decreasing time-pressure conditions (bottom row) in Experiment 1. Errors bars represent the standard error of the means.

Response times were analyzed first to ensure that participants complied with the task demands. The cross-subject means are illustrated in Figure 2 and show that observers’ average response times were within the time limits for each block. This was confirmed by an inspection of individual data. In the increasing pressure condition, for example, the slowest of all observers produced mean response times of 4.0, 2.7, 1.7, 2.0, and 1.5 seconds for the 10- to 2-second conditions, respectively. Similarly, in the decreasing pressure condition, the slowest of all observers produced mean response times of 2.9, 3.2, 3.7, 3.1, and 1.7 seconds for these conditions.

A 2 (Time Pressure: Increasing vs. Decreasing) × 2 (Trial Type: Match vs. Mismatch) × 5 (Time: 10, 8, 6, 4, and 2 seconds) mixed-factor analysis of variance (ANOVA) of the response time data revealed a three-way interaction, *F*(4,152) = 5.99, *p* < .001, $\eta_{\text{p}}^{2}$^ ^= 0.14. To analyze this interaction, separate 2 (Trial Type: Match vs. Mismatch) × 5 (Time: 10, 8, 6, 4, and 2 seconds) ANOVAs were conducted for the increasing and decreasing time-pressure conditions. For the increasing time-pressure condition, ANOVA did not show a main effect of trial type, *F*(1,19) = 3.03, *p* = .10, $\eta_{\text{p}}^{2}$^ ^= 0.14, but revealed a main effect of time, *F*(4,76) = 11.39, *p* < .001, $\eta_{\text{p}}^{2}$^ ^= 0.38, and an interaction between both factors, *F*(4,76) = 4.71, *p* < .01, $\eta_{\text{p}}^{2}$^ ^= 0.20. Analysis of simple main effects showed that response times for match and mismatch trials were comparable in the 10-second, *F*(1,19) = 3.91, *p* = .06, $\eta_{\text{p}}^{2}$^ ^= 0.17, 8-second, *F*(1,19) = 0.64, *p* = .43, $\eta_{\text{p}}^{2}$^ ^= 0.03, and 6-second condition, *F*(1,19) = 1.19, *p* = .29, $\eta_{\text{p}}^{2}$^ ^= 0.06. In contrast, responses were slower for mismatch than match trials in the 4-second, *F*(1,19) = 12.74, *p* < .01, $\eta_{\text{p}}^{2}$^ ^= 0.40, and 2-second condition, *F*(1,19) = 5.30, *p* < .05, $\eta_{\text{p}}^{2}$^ ^= 0.22. In addition, simple main effects of time were found for match,* F*(4,76) = 15.00, *p* < .01, $\eta_{\text{p}}^{2}$^ ^= 0.79, and mismatch trials, *F*(4,76) = 18.65, *p* < .01, $\eta_{\text{p}}^{2}$^ ^= 0.82. Bonferroni-adjusted pairwise comparisons showed that responses on match and mismatch trials were faster in the 2-second condition compared with all other conditions, all *p*s < .05. No other comparisons between conditions were significant, all *p*s ≥ .13.

For the decreasing time-pressure condition, ANOVA did not show a main effect of trial type,* F*(1,19) = 2.12, *p* < .16, $\eta_{\text{p}}^{2}$^ ^= 0.10, or an interaction between factors, *F*(4,76) = 2.41, *p* = .06, $\eta_{\text{p}}^{2}$^ ^= 0.11, but a main effect of time was found, *F*(4,76) = 7.57, *p* < .001, $\eta_{\text{p}}^{2}$^ ^= 0.29. Bonferroni-adjusted pairwise comparisons show that this arises due to faster response times in the 2-second compared with the 6- and 8-second conditions, both *p*s < .05, and in the 4-second than the 6-second condition, *p* < .05. No other comparisons reached significance, all *p*s ≥ .06.

### Accuracy

The data of most interest concern how accuracy was affected by the time limits. The cross-subject means of all observers’ percentage accuracy for the experimental conditions are shown in Figure 2. A 2 (Time Pressure: Increasing vs. Decreasing) × 2 (Trial Type: Match vs. Mismatch) × 5 (Time: 10, 8, 6, 4, and 2 seconds) mixed-factor ANOVA of these data revealed a three-way interaction, *F*(4,152) = 8.10, *p* < 0.001, $\eta_{\text{p}}^{2}$^ ^= 0.18. To analyze this interaction, separate 2 (Trial Type: Match vs. Mismatch) × 5 (Time: 10, 8, 6, 4, and 2 seconds) ANOVAs were conducted for the increasing and decreasing time-pressure conditions.

For the increasing time-pressure condition, ANOVA did not show a main effect of trial type, *F*(1,19) = 0.12, *p* = .74, $\eta_{\text{p}}^{2}$^ ^= 0.01, but revealed a main effect of time, *F*(4,76) = 4.51, *p* < .01, $\eta_{\text{p}}^{2}$^ ^= 0.19, and an interaction between these factors, *F*(4,76) = 4.65, *p* < .01, $\eta_{\text{p}}^{2}$^ ^= 0.20. Analysis of simple main effects did not find an effect of time for match trials, *F*(4,76) = 1.77, *p* = .18, $\eta_{\text{p}}^{2}$^ ^= 0.31, but showed such an effect for mismatch trials, *F*(4,76) = 6.25, *p* < .01, $\eta_{\text{p}}^{2}$^ ^= 0.61. For these mismatch trials, Bonferroni-adjusted pairwise comparisons revealed higher accuracy in the 10-second compared with the 2-second condition, *p* < .05. Mismatch accuracy was also higher in the 8-second condition than the 6-, 4-, and 2-second conditions, all *p*s < .05. No other comparisons reached significance, all *p*s ≥ .15. In addition, analysis of simple main effects also showed that accuracy was comparable for match and mismatch trials in the 10-, 8-, 6-, and 4-second conditions, all *F*s ≤ 1.83, *p*s ≥ 0.19, $\eta_{\text{p}}^{2}$^ ^≤ 0.09, but match accuracy was higher than mismatch accuracy in the 2-second condition, *F*(1,19) = 4.77, *p* < .05, $\eta_{\text{p}}^{2}$^ ^= 0.20.

For the decreasing time-pressure condition, a main effect of trial type was not found, *F*(1,19) = 0.18, *p* = .68, $\eta_{\text{p}}^{2}$^ ^= 0.01, but a main effect of time,* F*(4,76) = 4.67, *p* < .01, $\eta_{\text{p}}^{2}$^ ^= 0.20, and an interaction between factors, *F*(4,76) = 3.99, *p* < .01, $\eta_{\text{p}}^{2}$^ ^= 0.17. Analysis of simple main effects did not find an effect of time for mismatch trials, *F*(4,76) = 2.77, *p* = .06, $\eta_{\text{p}}^{2}$^ ^= 0.41, but showed such an effect for match trials, *F*(4,76) = 9.30, *p* < .001, $\eta_{\text{p}}^{2}$^ ^= 0.70. Bonferroni-adjusted pairwise comparisons showed that match accuracy was lower in the 2-second than the 8-second and 10-second conditions, both *p*s < .05, and lower in the 4- and 6-second conditions than the 10-second condition, both *p*s < .05. No other comparisons were significant, all *p*s ≥ .18. In addition, analysis of simple main effects also showed that accuracy was comparable for match and mismatch trials in all conditions, all *F*s ≤ 1.55, *p*s ≥ .23, $\eta_{\text{p}}^{2}$^ ^≤ 0.08, except the 10-second condition of Block 5, *F*(1,19) = 4.66, *p* < .05, $\eta_{\text{p}}^{2}$^ ^= 0.20.

### d′ and criterion

The percentage data were also converted into signal detection measures of sensitivity (*d′*) and response bias (*criterion*). For *d′*, a 2 (Time Pressure: Increasing vs. Decreasing) × 5 (Time: 10, 8, 6, 4, and 2 seconds) mixed-factor ANOVA did not find a main effect of time-pressure condition, *F*(1,38) = 0.33, *p* = .57, $\eta_{\text{p}}^{2}$^ ^= 0.01, but revealed a main effect of time, *F*(4,152) = 6.30, *p* < .001, $\eta_{\text{p}}^{2}$^ ^= 0.14, and an interaction between factors, *F*(4,152) = 4.85, *p* < .001, $\eta_{\text{p}}^{2}$^ ^= 0.11. Simple main effects of time were found for the increasing, *F*(4,152) = 7.02, *p* < .001, $\eta_{\text{p}}^{2}$^ ^= 0.45, and decreasing time-pressure conditions, *F*(4,152) = 7.76, *p* < .001, $\eta_{\text{p}}^{2}$^ ^= 0.44. Bonferroni-adjusted pairwise comparisons showed that sensitivity was lower in the 2- and 4-second conditions than the 8-second condition of the increasing time-pressure condition, both *p*s < .01, and lower in the 2-second condition than the 4-second and 10-second conditions of the decreasing time-pressure condition, both *p*s < .05. No other comparisons reached significance, all *p*s ≥ .17.

For *criterion*, a 2 (Time Pressure: Increasing vs. Decreasing) × 5 (Time: 10, 8, 6, 4, and 2 seconds) mixed-factor ANOVA did not find a main effect of time-pressure condition, *F*(1,38) = 0.06, *p* = .81, $\eta_{\text{p}}^{2}$^ ^= 0.00, or a main effect of time, *F*(4,152) = 0.92, *p* = .34, $\eta_{\text{p}}^{2}$^ ^= 0.02, but showed an interaction between factors, *F*(4,152) = 39.61, *p* < .001, $\eta_{\text{p}}^{2}$^ ^= 0.51. Again, simple main effects of time were found for the increasing, *F*(4,152) = 5.07, *p* < .001, $\eta_{\text{p}}^{2}$^ ^= 0.37, and decreasing time-pressure conditions, *F*(4,152) = 6.34, *p* < .001, $\eta_{\text{p}}^{2}$^ ^= 0.42. Bonferroni-adjusted pairwise comparisons showed that *criterion* was lower in the 2-second condition (Block 5) than the 10- and 8-second conditions (Blocks 1 and 2) under increasing time pressure, both *p*s < .01. Conversely, *criterion* was lower in the 10-second condition (Block 5) than the 2- and 4-second conditions (Blocks 1 and 2) under decreasing time pressure, both *p*s < 0.01. In addition, *criterion* was higher in the 10-second condition and lower in the 2-second condition under increasing compared with decreasing time pressure, both *p*s < .05. No other comparisons reached significance, all *p*s ≥ .32.

This pattern in *criterion* indicates that a response bias emerges during the course of the experiment, whereby participants become increasingly more likely to classify face pairs as identity matches. This effect is present irrespective of whether time pressure is increasing or decreasing during the task, indicating a separable effect. To confirm this effect, a series of one-sample *t* tests were conducted to compare *criterion* with zero. For the increasing time-pressure condition, this revealed a *criterion* that was reliably below zero in the 2-second condition of Block 5, *t*(19) = 2.35,* p* < .05, but not in any of the other time conditions, all *t*s(19) ≤ 1.21, all* p*s ≥ .24. Similarly, *criterion* was only reliably below zero in Block 5 of the decreasing time-pressure condition, *t*(19) = 2.50,* p* < .05, which corresponds to the 10-second time limit, but not in any of the other time conditions, all *t*s(19) ≤ 1.61, all* p*s ≥ 0.12.

### Time and Speed

In the current paradigm, time pressure was administered flexibly, so that lost time from slow responses on one trial (i.e., above the mean time limit for all trials in a block) could be compensated for by faster responses on other trials. To assess whether such compensatory behaviors affected accuracy, two further analyses were conducted. For the first analysis, the data were split into trials on which observers were on track to meet a time target (i.e., the mean of the response times for all trials completed in a block so far was below the mean time limit per trial for that block) or not (i.e., response times exceeded the time limit). This showed that observers exceeded the time target on only 0.54% of all trials (0.10% in the increasing and 0.97% in the decreasing time-pressure condition) and therefore yielded insufficient data points for further analysis.

The second analysis compared trials on which observers’ response times decreased compared with the immediately preceding trial, which could indicate a speed adjustment in an attempt to meet a time target, with trials on which response times increased. On average, observers were speeding up on 50.1% and slowing on 49.9% of trials, and overall accuracy appeared comparable across these conditions at 86.3% and 84.9%, respectively. Accordingly, a 2 (Speed: Speeding Up vs. Slowing Down) × 2 (Time Pressure: Increasing vs. Decreasing) × 2 (Trial Type: Match vs. Mismatch) × 5 (Time: 10, 8, 6, 4, and 2 seconds) mixed-factor ANOVA of these data did not find a main effect of speed, *F*(1,38) = 2.89, *p* = .10, $\eta_{\text{p}}^{2}$^ ^= 0.07, or an interaction of speed with any of the other factors, all *F*s ≤ 1.12, *p*s ≥ .35, $\eta_{\text{p}}^{2}$^ ^≤ 0.03.

## Discussion

This experiment examined the effect of time pressure on face matching accuracy, by systematically varying the available time for identification from 10 to 2 seconds across blocks. Time pressure was administered with a novel paradigm, which provided additional onscreen displays to indicate the number of faces that remained to be processed and informed observers as to whether they were on track to meet a time target for completing this task. When time pressure gradually increased during the experiment, by reducing the available average time for face matching from 10 to 2 seconds across blocks, an effect was found in response times, such that observers responded fastest in the 2-second condition. Importantly, the time-pressure manipulation also affected response accuracy. On match trials, accuracy was initially at 85% in the 10-second condition of Block 1, and remained stable across subsequent blocks. By contrast, accuracy decreased under time pressure on mismatch trials. This effect was most pronounced in Block 5, which allowed only 2 seconds on average for matching face pairs, in comparison to Block 1 (10 seconds) and Block 2 (8 seconds).

We compared this pattern with a second condition in which time pressure gradually decreased over the course of the experiment. This condition also revealed an effect in response times, such that observers responded fastest in the 2-second condition. Moreover, accuracy was lowest under the strictest time limit of 2 seconds. This is consistent with the increasing time-pressure condition and indicates an effect that operates independent of the order in which time pressure is administered. However, whereas match and mismatch accuracy was initially at 81% and 86% in the 2-second condition of Block 1, match accuracy increased over the course of the task to 95% in Block 5 but mismatch accuracy remained at 86%. Similar to the increasing time-pressure condition, analysis of signal detection measures indicates that this pattern reflects a response bias which developed over the course of the experiment, whereby participants were increasingly more likely to classify face pairs as identity matches.

Considered together, the data from the increasing and decreasing time-pressure conditions therefore reveal two separable effects on performance in this task. One effect reflects the time pressure that is administered in a block of trials, which can impair accuracy. This effect was observed particularly in the 2-second condition and was found regardless of whether this was administered at the beginning (Block 1 under decreasing time pressure) or end of the experiment (Block 5 under increasing time pressure). The second effect arises over the course of the experiment and presents as a response bias, whereby participants became increasingly more likely to classify face pairs as identity matches. This intriguing finding converges with recent reports of such a response bias in face-matching tasks and appears to reflect the prolonged or repetitive nature of this task (see Alenezi & Bindemann, 2013; Alenezi et al., 2015).

To provide further evidence for these effects, we conducted a second experiment in an attempt to replicate these findings. In this experiment, time pressure was administered by providing time limits of either 4 or 2 seconds in all five blocks on a between-subject basis. The advantage of this design is that time pressure cannot interact directly, on a block-by-block basis, with the response bias effect that emerges during the experiment. Thus, accuracy should be reduced generally in the 2-second compared with the 4-second condition, but observers should also develop a match bias over the course of the experiment in both of these time-pressure conditions.

A 4-second time limit was chosen as the closest comparison to the 2-second condition, which showed the greatest impairment in accuracy in the experiments so far. However, overall accuracy still exceeded 80% in the 2-second condition and observers were able to respond within the time limit. In Experiment 2, an additional time limit of just 1 second was therefore included. Our aim here was to determine the extent to which observers can still process face pairs correctly under such task demands.

## Experiment 2

In this experiment, participants matched pairs of faces under time limits of 1, 2, or 4 seconds. In contrast to the preceding experiment, these time limits were administered on a between-subject basis. Thus, each participant completed five blocks of 40 trials in only one of these conditions. The aim of this experiment is to provide further evidence that the time pressure and response bias effects are separable. We therefore predicted that accuracy would decline with time pressure, with worst performance in the 1-second condition. In addition, we expected to find a match bias that emerges over the course of the experiment in all conditions.

## Method

### Participants, Stimuli, and Procedure

Sixty new undergraduate students from the University of Kent (43 women), with a mean age of 19.8 years, participated for course credit or a small fee. All reported normal or corrected-to-normal vision. The stimuli and procedure were identical to the preceding experiment, except that the level of time pressure now remained constant at 1, 2, or 4 seconds across all five blocks of the experiment. Participants were allocated randomly to one of these three conditions.

## Results

### Response Times

**Figure 3.**
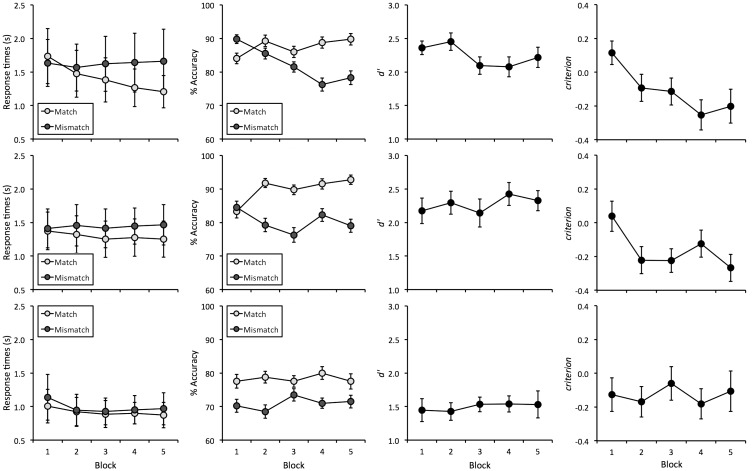
Response times (in seconds), face-matching accuracy (in %), *d′* and *criterion* for Experiment 2, for the 4-second condition (top row), the 2-second condition (middle row), and the 1-second condition (bottom row). Errors bars represent the standard error of the means.

The mean response times for all conditions are shown in Figure 3. These data indicate that these decreased across time-pressure conditions, with the fastest response times for 1-second displays. A 2 (Trial Type: Match vs. Mismatch) × 3 (Time Pressure: 4, 2, and 1 seconds) × 5 (Block: 1, 2, 3, 4, 5) mixed-factor ANOVA revealed a main effect of time pressure, *F*(2,57) = 12.73, *p* < .001, $\eta_{\text{p}}^{2}$^ ^= 0.31. Bonferroni-corrected pairwise comparisons showed that responses were generally slower in the 4- and 2-second conditions compared with the 1-second condition, both *p*s < .01, while the 4- and 2-second conditions did not differ, *p* = .57. In addition, a three-way interaction between factors was found, *F*(8,228) = 3.20, *p* < .01, $\eta_{\text{p}}^{2}$^ ^= 0.10. To interpret this interaction, three separate 2 (Trial Type) × 5 (Block) within-subjects ANOVAs were conducted for each time-pressure condition.

In the 4-second condition, a marginally significant main effect of trial type was found, *F*(1,19) = 4.23, *p* = .05, $\eta_{\text{p}}^{2}$^ ^= 0.18, a main effect of block, *F*(4,76) = 3.86, *p* < .01, $\eta_{\text{p}}^{2}$^ ^= 0.17, and an interaction between factors, *F*(4,76) = 4.31, *p* < .01, $\eta_{\text{p}}^{2}$^ ^= 0.19. Analysis of simple main effects showed that response times for match and mismatch trials were comparable in Block 1 and 2, all *F*s ≤ 2.24, *p*s ≥ .15, $\eta_{\text{p}}^{2}$^ ^≤ 0.11, but mismatch responses were slower than match responses in Blocks 3, 4, and 5, all *F*s(1,19) ≥ 5.03, *p*s < .05, $\eta_{\text{p}}^{2}$^ ^= 0.21. In addition, a simple main effect of block was found for match trials, *F*(4,76) = 4.37, *p* < .05, $\eta_{\text{p}}^{2}$^ ^= 0.52, but not for mismatch trials, *F*(4,76) = 0.36, *p* = .84, $\eta_{\text{p}}^{2}$^ ^= 0.08. Bonferroni-adjusted pairwise comparisons revealed faster response times for match trials in Block 4 and Block 5 compared with Block 1, both *p*s < .05. No other comparisons were significant, all *p*s ≥ .08.

For the 2-second condition, ANOVA showed no main effect of block, *F*(4,76) = 0.57, *p* = .69, $\eta_{\text{p}}^{2}$^ ^= 0.03, or two-way interaction, *F*(4,76) = 2.17, *p* = .08, $\eta_{\text{p}}^{2}$^ ^= 0.10, but a main effect of trial type was found, *F*(1,19) = 5.08, *p* < .05, $\eta_{\text{p}}^{2}$^ ^= 0.21, due to slower responses on mismatch trials.

Analysis of the 1-second condition also revealed an effect of trial type, *F*(1,19) = 6.15, *p* < .05, $\eta_{\text{p}}^{2}$^ ^= 0.25, due to slower responses on mismatch trials. A main effect of block was also found, *F*(4,76) = 3.11, *p* < .05, $\eta_{\text{p}}^{2}$^ ^= 0.14, but Bonferroni-adjusted pairwise comparisons failed to reveal differences between any of the blocks, all *p*s ≥ .11. The interaction between these factors was not significant, *F*(4,76) = 1.84, *p* = .13, $\eta_{\text{p}}^{2}$^ ^= 0.09.

### Accuracy

The mean percentage accuracy for all experimental conditions is shown in Figure 3. These data suggest that accuracy was better in the 4- and 2-second conditions compared with the 1-second group. In addition, match accuracy appears to increase in the 4- and 2-second conditions over the course of the experiment, but not in the 1-second group. A 2 (Trial Type) × 3 (Time Pressure) × 5 (Block) mixed-factor ANOVA of these data revealed a main effect of time pressure, *F*(2,57) = 11.68, *p* < .001, $\eta_{\text{p}}^{2}$^ ^= 0.29. Bonferroni-adjusted pairwise comparisons show that this arises from higher accuracy in the 2- and 4-second conditions compared with the 1-second condition, both *p*s < .01, whereas the 4-second and 2-second conditions did not differ, *p* = 1.00. In addition, a three-way interaction between all factors was found, *F*(8,228) = 2.16, *p* < .05, $\eta_{\text{p}}^{2}$^ ^= 0.07. To interpret this interaction, three separate 2 (Trial Type) × 5 (Block) within-subjects ANOVAs were conducted for each time-pressure condition.

For the 4-second condition, this analysis did not show a main effect of trial type, *F*(1, 19) = 2.09, *p* = .17, $\eta_{\text{p}}^{2}$^ ^= 0.10, but revealed an effect of block, *F*(4,76) = 2.78, *p* < .05, $\eta_{\text{p}}^{2}$^ ^= 0.13, and an interaction between factors, *F*(4,76) = 5.81, *p* < .001, $\eta_{\text{p}}^{2}$^ ^= 0.23. Analysis of simple main effects showed that accuracy was comparable for match and mismatch trials in Blocks 1, 2, and 3, all *F*s ≤ 2.95, *p*s ≥ .10, $\eta_{\text{p}}^{2}$^ ^≤ 0.13, whereas mismatch accuracy was marginally nonsignificantly lower than match accuracy in Block 5, *F*(1,19) = 3.96, *p* = .06, $\eta_{\text{p}}^{2}$^ ^= 0.17, and significantly lower than match accuracy in Block 4, *F*(1,19) = 6.30, *p* < .05, $\eta_{\text{p}}^{2}$^ ^= 0.25. In addition, simple main effects of block were found for match, *F*(4,76) = 4.98, *p* < .01, $\eta_{\text{p}}^{2}$^ ^= 0.56, and mismatch trials, *F*(4,76) = 3.56, *p* < .05, $\eta_{\text{p}}^{2}$^ ^= 0.47. Bonferroni-adjusted pairwise comparisons demonstrate a decrease in mismatch accuracy between Blocks 1 and 4, *p* < .05. No other comparisons for match or mismatch trials were significant, all *p*s ≥ .09.

In the 2-second condition, a main effect of block was not found, *F*(4,76) = 1.56, *p* = .20, $\eta_{\text{p}}^{2}$^ ^= 0.08, but a main effect of trial type, *F*(1,19) = 6.67, *p* < .05, $\eta_{\text{p}}^{2}$^ ^= 0.26, and an interaction between factors, *F*(4,76) = 5.46, *p* < .01, $\eta_{\text{p}}^{2}$^ ^= 0.22. Analysis of simple main effects showed that accuracy was comparable for match and mismatch trials in Block 1, *F*(1,19) = 0.07, *p* = .80, $\eta_{\text{p}}^{2}$^ ^= 0.00, whereas mismatch accuracy was lower than match accuracy in Blocks 2, 3, 4, and 5, all *F*s ≥ 4.45, *p*s ≤ .05, $\eta_{\text{p}}^{2}$^ ^≥ 0.19. In addition, marginally nonsignificant simple main effects of block were found for match trials, *F*(4,76) = 2.89, *p* = .06, $\eta_{\text{p}}^{2}$^ ^= 0.42, and mismatch trials, *F*(4,76) = 2.83 *p* = .06, $\eta_{\text{p}}^{2}$^ ^= 0.42. Bonferroni-adjusted pairwise comparisons showed that match accuracy increased between Blocks 1 and 5, *p* < .05. No other comparisons for match or mismatch trials were significant, all *p*s ≥ .06.

Finally, in the 1-second condition, no main effects of trial type, *F*(1,19) = 2.20, *p* = .15, $\eta_{\text{p}}^{2}$^ ^= 0.10, and block, *F*(4,76) = 0.26, *p* = .90, $\eta_{\text{p}}^{2}$^ ^= 0.01, or an interaction were found, *F*(4,76) = 0.41, *p* = .80, $\eta_{\text{p}}^{2}$^ ^= 0.02.

### d' and criterion

As in the previous experiment, percentage accuracy scores were also converted into *d*′ and *criterion* (see Figure 3). For *d*′, a 3 (Time Pressure) × 5 (Block) mixed-factor ANOVA did not reveal a main effect of block, *F*(4,228) = 0.70, *p* = .59, $\eta_{\text{p}}^{2}$^ ^= 0.01, or an interaction of block and time pressure, *F*(8,228) = 1.69, *p* = .10, $\eta_{\text{p}}^{2}$^ ^= 0.06, but an effect of time pressure was found, *F*(2,57) = 12.52, *p* < .001, $\eta_{\text{p}}^{2}$^ ^= 0.31. Bonferroni-adjusted pairwise comparisons revealed that sensitivity was lower in the 1-second compared with the 2- and 4-second conditions, both *p*s < .001, whereas the 2- and 4-second conditions did not differ from each other, *p* = 1.00.

A 3 (Time Pressure) × 5 (Block) ANOVA of *criterion* did not reveal a main effect of time pressure, *F*(2,57) = 0.13, *p* = .88, $\eta_{\text{p}}^{2}$^ ^< 0.01, but showed a main effect of block, *F*(4,228) = 6.12, *p* < .001, $\eta_{\text{p}}^{2}$^ ^= 0.10, and an interaction between factors, *F*(8,228) = 2.56, *p* < .05, $\eta_{\text{p}}^{2}$^ ^= 0.08. Analysis of simple main effects revealed an effect of block in the 4-second condition, *F*(4,154) = 4.13, *p* < .01, $\eta_{\text{p}}^{2}$^ ^= 0.23, and the 2-second condition,* F*(4,154) = 3.73, *p* < .01, $\eta_{\text{p}}^{2}$^ ^= 0.22, but not the 1-second condition, *F*(4,154) = 1.20, *p* = .32, $\eta_{\text{p}}^{2}$^ ^= 0.08. For the 4-second condition, Bonferroni-adjusted pairwise comparisons revealed a criterion shift from Block 1 to Blocks 4 and 5, both *p*s < .05, which indicates that participants developed a bias to make more match responses over the course of the experiment. Similarly, the 2-second condition revealed a criterion shift to make more match responses when Block 1 was compared with Blocks 2, 3, and 5, all *p*s < .05. No other comparisons were significant, all *p*s ≥ .07.

Once again, a series of one-sample *t* tests were also conducted to compare *criterion* to zero. This revealed that *criterion* was reliably below zero in Block 4 of the 4-second condition, *t*(19) = 2.86,* p* < .05, and in Blocks 2, 3, and 5 of the 2-second condition, all *t*s(19) ≥ 2.95, all* p*s < .01. No other comparisons in any of the time-pressure conditions reached significance, all *t*s(19) ≤ 2.01, all* p*s ≥ .05.

### Time and Speed

As in Experiment 1, we assessed accuracy as a function of whether participants were ahead or behind the target time. In the 4- and 2-second conditions, participants’ responses exceeded the target time on only a small proportion of trials (corresponding to 0% and 2.5% for these conditions, respectively), yielding insufficient data for further analysis. In the 1-second condition, response times for 25.7% of trials fell outside of the target time limit. A 2 (Time Target: Ahead vs. Behind) × 2 (Trial Type: Match vs. Mismatch) ANOVA of these data did not reveal a main effect of trial type, *F*(1,16) = 3.16, *p* = .09, $\eta_{\text{p}}^{2}$^ ^= 0.17, or of meeting the time target, *F*(1,16) = 0.76, *p* = .40, $\eta_{\text{p}}^{2}$^ ^= 0.05, or an interaction between factors, *F*(1,16) = 1.10, *p* = .31, $\eta_{\text{p}}^{2}$^ ^= 0.06.

Once again, a second analysis also compared the trials on which observers’ response times decreased or increased compared with the immediately preceding trial. On average, observers were speeding up and slowing down on a comparable number of trials across conditions (4 seconds: 49.9% vs. 50.1%; 2 seconds: 49.9% vs. 50.1%; 1 second: 49.7% vs. 50.3%, respectively), yielding sufficient trials for further analysis. A 2 (Speed: Speeding Up vs. Slowing Down) × 2 (Trial Type: Match vs. Mismatch) × 3 (Time Pressure: 4, 2, and 1 seconds) mixed-factor ANOVA of these data revealed a main effect of trial type, *F*(1,57) = 8.47, *p* < .01, $\eta_{\text{p}}^{2}$^ ^= 0.13, due to higher accuracy for match than mismatch trials (85.6% vs. 78.7%), a main effect of time-pressure condition (4 seconds 85.6% vs. 2 seconds 85.4% vs. 1 second 75.5%), *F*(2,57) = 10.55, *p* < .001, $\eta_{\text{p}}^{2}$^ ^= 0.27, and an interaction of speed and time pressure condition, *F*(2,57) = 4.30, *p* < .05, $\eta_{\text{p}}^{2}$^ ^= 0.13. Bonferroni-adjusted pairwise comparisons revealed that accuracy was lower in the 1-second condition on speeding up (73.8%) than slowing down trials (77.1%), *p* < .05 (4-second condition, 86.7% vs. 84.4%; 2-second condition, 86.0% vs. 84.8%). In addition, on both speeding up and slowing down trials, accuracy was lower in the 1-second than the 2- and 4-second conditions, all *p*s < .01. None of the other comparisons, all *p*s ≥ .11, the main effect of speed, *F*(1,57) = 0.02, *p* = .90, $\eta_{\text{p}}^{2}$^ ^= 0.00, or the remaining interactions were significant, all *F*s ≤ 0.84, all* p*s ≥ .36, $\eta_{\text{p}}^{2}$^ ^≤ 0.01.

## Discussion

This experiment revealed an effect of time pressure, whereby accuracy was worst for the 1-second compared with the 2- and 4-second conditions. In numerical terms, this effect accounted for approximately 10% of errors and was observed in a context in which observers could complete the task within a 2- and 4-second limit but struggled to stay within the 1-second target. This effect was such that 6 out of 20 observers failed to meet this time target over the duration of the entire experiment, and 8 out of 20 observers could not stay within this time target in at least one of the blocks. Overall, these data therefore indicate that a 1-second time limit compromises face matching.

These data were analyzed further to assess if accuracy was affected by whether participants were ahead of or behind the target time. In the 4- and 2-second conditions, the target time was exceeded rarely, which yielded insufficient trials for analysis. In the 1-second condition, on the other hand, responses fell outside of the target time on one in four trials. However, accuracy was not impaired in these instances. This pattern makes good sense considering the flexible administration of time pressure that formed the premise for the current experiments. We reasoned a priori that observers may require additional time in some instances, and less time in others, to reach a correct identification decision. By providing such an allowance through the flexible administration of time limits, accuracy should be similar for slower matching decisions and those that can be decided more quickly.

This framework rests on the assumption that any lost time can be recouped through the speeding up of subsequent responses. However, in Experiment 2, accuracy was also reduced on subsequent trials with such faster response times. This could suggest that participants were attempting to compensate in speed for slower responses, which then impaired performance. Generally, however, this effect was only observed in the 1-second condition and was equivalent to a 3.3% decrease in accuracy, which indicates a numerically small effect.

In addition to the time-pressure effect, the 2- and 4-second conditions also revealed a response bias, such that observers made more match responses during the latter blocks of the task. This finding converges with the results of Experiment 1 to suggest that the effect of time pressure, which reduces accuracy generally, is separable here from a response bias, which increases the likelihood that match decisions are made. In contrast to the preceding experiment, which demonstrated this effect under increasing and decreasing time pressure, this effect was observed in Experiment 2 with a design in which the mean time limit remained constant across blocks.

Finally, we note that such a bias was not observed in the 1-second condition. In this condition, a match bias was already apparent in Block 1 and was maintained throughout the experiment. However, this effect was not reliable. We suggest that the formation of a response bias across blocks was prevented or obscured by the generally reduced performance in this condition.

## General Discussion

This study examined the effect of time pressure on face-matching accuracy. For this purpose, two onscreen displays provided information about the numbers of faces that still needed to be matched in the experiment and whether observers were on track to complete this task in a given time frame. In both experiments, an effect of time pressure on performance was found, but this differed in expression. For example, *d*′ decreased significantly from the 8-second condition (Block 2) to the 4- and 2-second conditions (Blocks 4 and 5) under increasing time pressure in Experiment 1, whereas this sensitivity measure was lower in the 2-second condition (now Block 1) compared with the 4- and 10-second conditions (Blocks 2 and 5) under decreasing time pressure. In addition, *d*′ was reduced in the 1-second condition of Experiment 2 compared with the 2- and 4-second conditions. Overall, these experiments therefore converge to show that time pressure impairs face-matching performance, though it is difficult to specify a precise cutoff at which this falls off consistently.

The pattern of response times also varied in the precise expression of effects across conditions and experiments. For example, under increasing time pressure in Experiment 1, response times were consistent across the 10- to 4-second conditions and decreased in the 2-second condition. Under decreasing time pressure, on the other hand, response times increased from the 2- to the 4- and 6-second conditions, and remained constant thereafter. In addition, response times decreased across blocks in the 4-second match, but not mismatch, condition of Experiment 2, but remained more stable across blocks in the 2- and 1-second conditions.

At present, we cannot adequately explain the variability in these accuracy and response time patterns across conditions and experiments. However, it is likely to reflect, at least in part, the differences in experimental design (e.g., increasing, decreasing, or constant time pressure). We also note that the overall effect of time pressure on face-matching accuracy was generally numerically small. For example, in the increasing time-pressure condition of Experiment 1, accuracy varied from 87% in the 10-second condition to 84% in the 2-second condition, and from 90% to 83% when time pressure was decreasing. Similarly, overall accuracy was at 85% for the 4- and 2-second conditions of Experiment 2, but only decreased to 75% with a mean time limit of 1 second.

Some other aspects of our novel paradigm also exerted only relatively small effects on accuracy. A key premise of this paradigm was that previous studies administered time pressure on a trial-by-trial basis (Lee et al., 2006; Özbek & Bindemann, 2011; White, Phillips, et al., 2015), whereas such pressure applies to more extended periods in occupational settings. We therefore created conditions that allow observers additional time on some trials to make an identification, which could be compensated for by faster responses on other trials. In this framework, the flexible administration of time limits should produce similar accuracy for slower and faster identifications. Our results indicate as much, by revealing comparable accuracy for trials on which observers were ahead of or behind the target time. We also assessed whether accuracy is affected by increases in response speed that might reflect compensation for slower responses on previous trials. This analysis showed that accuracy varied only by 3% as a function of response speed and this was observed only in the strictest 1-second time limit condition of Experiment 2.

Overall, the current findings indicate that time pressure exerts relatively modest effects on face-matching accuracy, unless very strict time limits of less than 2 seconds are imposed (see Experiment 2 and Özbek & Bindemann, 2011). We note, however, that these effects were observed under idealized conditions, with an established stimulus set that yields comparatively high accuracy (see Burton et al., 2010). This raises the possibility that this test is not particularly sensitive to detect time-pressure effects and could account for the inconsistencies across the increasing, decreasing, and constant time-pressure conditions. This issue is compounded by the consistently fast response times in all conditions across the experiments. For example, although accuracy was higher in the 8-second condition compared with the 4- and 2-second conditions under increasing time pressure in Experiment 1, mean response times in all of these conditions were below the most stringent time limit of 2 seconds.

Considering that relevant information was available throughout the experiment from the queue and speed displays, participants’ reluctance to use all of the available time might converge with the notion that the current stimuli were not sufficiently difficult to elicit clearer time-pressure effects. Alternatively, it is possible that our student sample was not motivated to make full use of the time limits, as this would have resulted in additional time on the task. In line with this reasoning, it has recently been shown that both forensic expert face examiners and nonexpert government employees outperform student participants in face matching, which might be indicative of motivational effects (White, Phillips, et al., 2015). However, there is also evidence that passport officers take substantially longer than student participants to make matching decisions but without an accuracy advantage (White et al., 2014). Further studies are clearly needed to assess the effect of time pressure further, encompassing more taxing stimuli than in the experiments reported here and different populations. The current paradigm, which allows for trial-by-trial flexibility in the application of time limits, provides a good basis for such research.

The current study revealed another factor that appears to affect face-matching accuracy, as a response bias developed during the course of the task whereby observers were increasingly likely to classify face pairs as identity matches. This effect varied somewhat in its expression across conditions. In the increasing time-pressure condition of Experiment 1, for example, match accuracy remained stable across blocks but mismatch accuracy declined. The reverse pattern was evident under decreasing time pressure, with stable mismatch accuracy and increasing match accuracy across blocks. The cause of these subtle differences is not yet clear. However, both the increasing and decreasing time-pressure conditions of Experiment 1, and the 2- and 4-second conditions of Experiment 2, revealed a clear and consistent match bias when accuracy was converted into *criterion*.

A similar effect has been observed recently in two other studies, which demonstrate a match bias that increases continuously over 1,000 trials (Alenezi & Bindemann, 2013; Alenezi et al., 2015). In these studies, this effect is already discernible over the first 200 trials, which converges with the pattern observed here. This effect is not alleviated by regular rest breaks or context switches, which indicates that it does not reflect fatigue or habituation to task goals (see Alenezi et al., 2015). This might suggest that the cause of this bias is not cognitive but perceptual in origin. However, this notion is also difficult to reconcile with the finding that this bias can be eliminated through the administration of task feedback (Alenezi & Bindemann, 2013).

Irrespective of the cause of the match bias, the discovery and replication of this effect might be an important finding for occupational settings in which observers have to match faces routinely over long intervals, such as person identification at passport control. In these settings, the detection of identity mismatches, or impostors, is of particular importance (see CPNI, 2007; FRONTEX, 2012; Stevens, 2011). The finding that a match bias, which reflects a specific difficulty in detecting such mismatches, develops during this task suggests that the detection of impostors is particularly compromised. The current experiments also demonstrate that accuracy can be impaired by time pressure, even when this is applied flexibly so that particularly slow responses on some trials can be compensated for on others, and when highly optimized stimuli are employed. These findings raise further concern about person identification in security settings.
